# Emerging SARS-CoV-2 Variants of Concern (VOCs): An Impending Global Crisis

**DOI:** 10.3390/biomedicines9101303

**Published:** 2021-09-23

**Authors:** Angel Yun-Kuan Thye, Jodi Woan-Fei Law, Priyia Pusparajah, Vengadesh Letchumanan, Kok-Gan Chan, Learn-Han Lee

**Affiliations:** 1 Novel Bacteria and Drug Discovery Research Group (NBDD), Microbiome and Bioresource Research Strength (MBRS), Jeffrey Cheah School of Medicine and Health Sciences, Monash University Malaysia, Subang Jaya 47500, Malaysia; angelthye.yunkuan@monash.edu (A.Y.-K.T.); jodi.law1@monash.edu (J.W.-F.L.); priyia.pusparajah@monash.edu (P.P.); 2 Division of Genetics and Molecular Biology, Institute of Biological Sciences, Faculty of Science, University of Malaya, Kuala Lumpur 50603, Malaysia; 3 International Genome Centre, Jiangsu University, Zhenjiang 212013, China

**Keywords:** SARS-CoV-2, mutations, variant of concern (VOC), transmissibility, progenitor

## Abstract

The worldwide battle against the SARS-CoV-2 virus rages on, with millions infected and many innocent lives lost. The causative organism, severe acute respiratory syndrome coronavirus 2 (SARS-CoV-2), is a beta coronavirus that belongs to the Coronaviridae family. Many clinically significant variants have emerged, as the virus’s genome is prone to various mutations, leading to antigenic drift and resulting in evasion of host immune recognition. The current variants of concern (VOCs) include B.1.1.7 (Alpha), B.1.351 (Beta), B.1.617/B.1.617.2 (Delta), and P.1 (Gamma). The emerging variants contain various important mutations on the spike protein, leading to deleterious consequences, such as immune invasion and vaccine escape. These adverse effects result in increased transmissibility, morbidity, and mortality and the evasion of detection by existing or currently available diagnostic tests, potentially delaying diagnosis and treatment. This review discusses the key mutations present in the VOC strains and provides insights into how these mutations allow for greater transmissibility and immune evasion than the progenitor strain. Continuous monitoring and surveillance of VOC strains play a vital role in preventing and controlling the virus’s spread.

## 1. Introduction

In 2020, a global pandemic caused by the severe acute respiratory syndrome coronavirus 2 (SARS-CoV-2) struck. The original virus strain was first discovered in Wuhan, Hubei province of China [1,2,3,4,5], but it spread rapidly across the globe, spawning several mutations, and by April 2020, the SARS-CoV-2 variant carrying the spike protein amino acid D614G mutation became the dominant form worldwide [6]. The virus genome was sequenced and postulated to be of zoonotic origin, with the most likely origin being bats [7,8]. However, the intermediate host of the SARS-CoV-2 is yet to be confirmed [9]. Although the evidence suggested that the spread of the virus was waning by the end of 2020, the persistence of SARS-CoV-2 has enabled it to mutate and incessantly cause new waves of infection in many countries. At the time this review went to press, a total of 195,617,174 confirmed cases with over 4 million deaths were reported globally (as of 29 July 2021). Despite the development of vaccines and the global initiation of immunization, the incessant rise in the number of cases globally reflects the impact of new variants of SARS-CoV-2. The variants of concern (VOCs) are variants with evolutionary advantages that are favored [10,11]. These new variants emerged around the same time in multiple locations that were independent of one another, starting from September 2020. It began with the emergence of B.1.1.7 in the United Kingdom (UK) [12], then B.1.351 in South Africa [13], followed by B.1.617 in India [14] and P.1 in Brazil [15]. These new variants have multiple mutations on their spike protein and spread rapidly across the globe in a short time, suggesting that they are more virulent.

Mutations in the spike protein are of particular concern, as vaccines were mainly designed to trigger the generation of antibodies against components of the spike protein. The spike protein antigens selected to evoke T cell responses against SARS-CoV-2 were developed based on spike sequences derived from the progenitor strain and involved RNA, recombinant protein, virally vectored platforms, and inactivated virus [16]. Following rapid trials, within about ten months from publication of the first sequence of SARS-CoV-2, vaccine efficacy results were readily available to the public [17,18,19]. Pharmaceutical companies raced against time to produce and distribute vaccines across the globe. Pfizer, Moderna, Sinovac, and AstraZeneca vaccines are among the few vaccines approved for emergency use by the World Health Organization (WHO) and have been administrated to millions of people globally [20]. Nevertheless, reports have shown that the currently available vaccines are ineffective against the VOC strains, thus prompting consideration of booster immunizations.

The emergence of VOCs is concerning, as these mutations may affect global epidemiology due to their high transmissibility, becoming the predominant strain in those countries affected, and could cause uncertainties in vaccine efficacy due to their potential in immune evasion. Although the efficacy of current vaccines seems to be less affected by the B.1.1.7 variant [21,22], there is a reduction in vaccine efficacy against the B.1.351 variant [23,24,25,26], while studies on the effectiveness against the B.1.617/B.1.617.2 variant are still ongoing. Hence, this study discusses the key mutations present in the VOC strains and provides insights into how these mutations allow for greater transmissibility and immune evasion compared with the progenitor strain.

## 2. Variants of Concern (VOCs)

A theory that has been proposed regarding the simultaneous emergence of mutated spike protein at multiple different locations is that prolonged infection in immunocompromised hosts might drive antigenic evolution [27]. In those with persistent infections, recurrent deletions in the spike protein occur as the virus replicates, resulting in variations resistant to neutralizing antibodies [28]. This trend of viral gene deletions occurring in infected immunocompromised patients could possibly increase the severity of COVID-19 by causing the evolution of variant strains that possess adaptive features such as higher infectivity and immune evasion capabilities [28,29]. In addition, convalescent plasma treatment in immunocompromised patients with COVID-19 infection may also correlate with new viral variants resulting from selective pressure. A study showed that the transient emergence of amino acid 69 and 70 (Δ69–70) deletion and N501Y mutation in B.1.1.7 may have resulted from the repeated use of plasma therapy in an immunocompromised individual [29]. The published data revealed that a VOC becomes the main strain in the region where it is first detected and spreads globally. A summary of the characteristics of VOC strains is presented in Table 1.

### 2.1. B.1.1.7 (Alpha)

The B.1.1.7 strain, referred to as the Alpha variant, was first detected in England on 20 September 2020. The number of people infected with this strain increased exponentially, and by February 2021, the Alpha variant accounted for nearly 95% of SARS-CoV-2 transmission in England [30]. Regarding the demographic effect on SARS-CoV-2, a study in the southeast of France demonstrated that B.1.1.7 affects mainly healthier and younger patients [31]. However, another study showed that the proportion of cases in younger age groups (<60 years) was similar for non-VOC and VOC cases, with similar mean ages for B.1.1.7 [32].

As compared with the progenitor strain, B.1.1.7 consists of nine mutations on the SARS-CoV-2 spike protein, involving two deletions and seven amino acid substitutions (four in S1 and three in S2): Δ69–70HV and Δ144Y deletions; N501Y, D614G, A570D, P681H, T716I, S982A, and D1118H [33]. In addition, four out of the seven spike protein substitutions are found occurring at the intermolecular interaction site, allowing B.1.1.7 to be distinguished from the progenitor strain [34]. According to the CDC, mutations E484K, S494P, and K1191N are also present but only to be found in some sequences [35]. Mutations found on B.1.1.7 that will be discussed more in this paper are mainly on the N501Y, P681H, A570D, D614G, S982A, Δ144Y, and Δ69–70HV deletions. These mutations relate to the increase in binding affinity, cell entry, infectivity, and neutralization that is characteristic of the VOC.

The B.1.1.7 has higher transmissibility (43–82% more transmissible) [30], higher viral load [23,36], longer duration of infection [26], higher hospitalization rate [32], and higher mortality rate [25]. The B.1.1.7 has been reported to have higher reproduction numbers— a rate 40–90% higher than D614G. The enhanced transmissibility results from N501Y substitution, which boosts the accessibility of receptor-binding domain (RBD) and affinity with host cell receptors, leads to better viral establishment and infection propagation [24,37]. In terms of higher viral load in patients with VOCs, there are conflicting data showing no significant difference between the B.1.1.7 variant and non-VOCs [38]. Furthermore, it is found that B.1.1.7 cases involving patients <60 years old have a higher hospitalization rate and admission rate to an intensive unit care than non-VOC cases [32]. This is consistent with two other reports, one from Denmark [39] and one from Germany. As compared with the previous variants, it is estimated that the risk of mortality of B.1.1.7 rose from 2.5 to 4.1 deaths per 1000 cases [25,40] and has an average infection lasting 13.3 days instead of the 8.2 days seen in other variants [41]. Again, there are variable reports of severity, as some studies have shown that B.1.1.7 does not cause an increase in severity and has a loss-of-function mutation in Orf8 associated with milder disease [37], consistent with other controlled studies [42,43].

B.1.1.7 is resistant to neutralization by most of the monoclonal antibodies (mAbs) directed against the N-terminal domain (NTD) and slightly resistant to some mAbs directed against the RBD. However, it is less resistant to the serum of SARS-CoV-2 vaccinated individuals and plasma of recovered SARS-CoV-2 patients [44]. That being said, the reinfection of B.1.1.7 is not found to be higher than other strains [42], and it is still susceptible to neutralizing antibodies by spike vaccines [21,22]. This is consistent with two studies stating that the mutation of N501Y in B.1.1.7 does not lead to antibody evasion and thus should not affect vaccine-induced neutralization [45,46]. Nonetheless, there are debates about whether a slight decrease in vaccine efficacy is present or if it is the result of a small sample size [47].

### 2.2. B.1.351 (Beta)

B.1.351 or the Beta variant likely emerged from the first wave of the Covid-19 epidemic in South Africa, which hit the Nelson Mandela Bay metropolitan area in the Eastern Cape Province in October 2020, rearing its head just after a drop in the first wave. By the end of November 2020, it spread across Western and Eastern Cape Provinces and became the predominant virus lineage [13]. By January 2021, the B.1.351 variant had spread to several other countries, including Botswana, Scotland, England, France, Switzerland, Sweden, South Korea, and Australia [48]. The B.1.351 is characterized by 18 amino acid mutations (7 in the spike protein) and 3 amino acid deletions in the spike protein [49]. According to Tegally et al., relative to the progenitor sequence, the B.1.351 viral isolate consists of 10 changes: replacements of A701V, D215G, D80A; D614G spike mutation; 3 RBD mutations at E484K, K417N, N501Y; amino acid L242–244 deletions and R246I and L18F [50].

As compared with the progenitor strain, B.1.351 has higher transmissibility [13], viral load [49], reinfection, vaccine escape [51], hospitalization rate [32], and mortality [52]. A modeling study estimated that B.1.351 is approximately 50% more transmissible than other circulating variants [53]. This is seen in the spread of B.1.351 through Western Cape in South Africa, where it reached 100,000 cases 50% more rapidly than the first SARS-CoV-2 wave. The increased transmissibility is caused by the N501Y mutation in the RBD of the spike protein [52], which causes an increased affinity for ACE2 [54]. B.1.351 is resistant to neutralization by the majority of mAbs directed against the NTD and many mAbs directed against the RBD due to E4848K substitution. It is also more resistant to neutralization by sera from vaccinated individuals and convalescent plasma [44]. It also poses a high risk of reinfection, as it is highly resistant to neutralizing antibodies formed by being infected by previously circulating lineages [51]. Furthermore, compared with non-VOC cases, B.1.351 cases involving those <60 years old were found to have a higher hospitalization rate and admission rate to intensive unit care [32]. Compared with the first COVID-19 wave, South Africa’s in hospital mortality rose 20% in the second COVID-19 wave due to increased transmissibility and not increased severity, as the healthcare system was overburdened, which affected hospital care [52].

Hypotheses are suggesting that B.1.351 originated via an intra-host viral evolution because of prolonged infection in immunocompromised hosts [27]. Although the spontaneous mutations in E484K and N501Y were reported after 75 and 128 days of infection, other mutations require contributions from other viral lineages, making this hypothesis only partially valid [13]. Mutations of great concern in B.1.351 are at the RBD: N501Y, E484K, and K417N [13,55]. These mutations relate to the increase in binding affinity, cell entry, infectivity, and resistance to neutralization.

### 2.3. B.1.617/B.1.617.2 (Delta)

B.1.617 consists of three sub-lineages: B.1.617.1, B.1.617.2, and B.1.617.3. The B.1.617.1 and B.1.617.2 sub-lineages were first detected in India in December 2020, whereas the B.1.617.3 sub-lineage was first detected in India in February 2021 [56]. B.1.617 emerged from India, with its first reported samples collected from Maharashtra, India, in October 2020. It rapidly became the dominant variant in India. A study by the National Institute of Virology in Pune found that >60% of samples collected in the state from January to March were of the B.1.617 variant [57].

Regarding demographics, the second wave in India involved more pediatric cases. The number of cases in the first wave in India was around 10–15% of the caseload of those seen in the second wave. Between 1 January and 21 April, government data showed that 11.5% of the 5.6 million COVID-positive cases were between 0 and 20 years old [58]. There are also reports stating that pediatric cases rose in all cities with severe epidemics [59,60]. Research indicates that B.1.617.2 is 60% more transmissible than B.1.1.7 and has a higher risk of hospitalization [61]. The emergence of B.1.617 that resulted in an exponential increase in COVID cases could be due to mutations, densely populated cities, a lack of testing, and an insufficiency in genomic testing, which then impacts disease management and the system [62]. This consequently led some countries to ban travel from India.

According to the CDC, B.1.617.1, known as the Kappa variant or 20A/S:154K, is characterized by multiple mutations: P681R, E154K, L452R, E484Q, G142D, D614G, Q1071H, and T951. The T951 is found only in some B.1.617.1; B.1.617.2, also known as the Delta variant or 20A/S:478K, has spike mutations: D614G, D950N, L452R, T19R, T478K, P681R, 156–157 deletion and R158G and G142D. The G142D is found only in some B.1.617.2; B.1.617.3, with the next strain name 20A, has spike protein mutations: D614G, D950N, L452R, P681R, T19R, E484Q, and G142D [62]. Currently, data associated with B.1.617 are still insufficient. Still, experiments with B.1.617.1 in the hamster model point to a higher viral load in the lungs and higher pathogenicity than the D614G variant [63]. However, no mutation in any of the B.1.617 variant subtypes is linked with increased disease severity [64]. The E484Q found in B.1.617.1 is associated with vaccine escape, while the L452R and T478K found in B.1.617.2 is associated with vaccine escape and increased transmissibility [64]. Mutations that are of interest and that will be discussed in this paper are the L452R, E484Q, and P618R. These mutations impact binding affinity, infectivity, immune evasion, and resistance to neutralization.

### 2.4. P.1 (Gamma)

P.1, referred to as the Gamma variant, is a descendent of the B.1.1.28 lineage, first detected in early March 2020 [65]. P.1 is not given the designation B.1.1.28.1 but is given a new lineage designation because P.1 is genetically and phylogenetically distinct from ancestral viruses and is linked with an accelerated spread in a new area—Manaus, Brazil. Furthermore, it has mutations that may have functional and/or phenotypic relevance [66]. The first wave of infection in Manaus started in March–June 2020, and by October, about 75% of individuals had been infected [67]. The emergence of the P.1 lineage marks the beginning of the second wave in Manaus. The P.1 variant was first detected on the 6 December 2020 in Manaus, Amazonas State, northern Brazil [65]. It is also linked to travel-related cases detected in Japan [68] and São Paulo [65]. According to evolutionary clock analysis, this variant emerged a month before being detected in the middle of November 2020, 3 to 4 weeks before the resurgence in SARS-CoV-2 confirmed cases in Manaus. Within two months, between 2 November 2020 and 9 January 2021, Manaus had 7137 severe acute respiratory infection (SARI) cases and 3144 SARI deaths. In January 2021, P.1 corresponded to 87% of all infections in Manaus [65]. The P.1 variant contains multiple spike protein mutations, including K417T, E484K, N501Y in the RBD; L18F, T20N, P26S, D138Y, R190S in the NTD; D614G and H655Y at C terminus in S1; and V1176F and T1027I in S2 [67]. The biologically important mutations in P.1 include the N501Y, E484K, and K417T.

This variant can evade the immune system, as it is resistant to neutralization by some RBD-directed mAbs, including three with EUA [69], mainly due to E484K [51,70,71,72]. As of March 2021, Brazil had reported >13 million SARS-CoV-2 cases and recorded >300,000 deaths [65]. It is still uncertain whether the P.1 variant that circulated during the second wave is associated with higher transmissibility than previous lineages found in Manaus [73]. The P.1 variant does share some independently acquired mutations with B.1.1.7 (N501Y) and B.1.351 (N501Y, E484K, and K417N/T), which have been associated with higher transmissibility [13]. Moreover, a preprint study using a model-based approach estimated that the transmissibility of P.1 is about 2.5 times higher than previous variants in Manaus and that it has a probability of reinfection of 6.4% [74]. This is consistent with two other studies that used different approaches. Based on the phylogenetic method, one study estimated the effective reproduction number to be 2.2 times higher for the P.1 variant, hypothesizing at least a 2-fold increase in transmissibility compared with the parental lineage, presuming reinfections as uncommon events [75]. Another study using a semi-mechanistic Bayesian model estimated P.1 to be 1.7–2.4 times more transmissible than previously circulating strains and to evade 21–46% of the protective immunity in individuals once infected with a non-P.1 variant, as well as to have a 54–79% cross-immunity [65]. Higher viral load was also found in a P.1-associated reinfection case detected in Manaus [15]. In terms of hospitalization, from November 2020, a study found an increase in hospitalization and P.1 clinical samples [74]. Another study also showed that in comparison with non-VOC cases, there was a higher hospitalization rate and admission rate to an intensive unit care for P.1 cases, especially for those <60 years of age. However, in terms of the disease severity of P.1, there are a lack of published data [32]. Mortality seems to increase by 1.2- to 1.9-fold, but it could be confounded by the health care capacity in Manaus [65].

**Table 1:** A summary of the characteristics of variants of concern (VOCs).

	B.1.1.7	B.1.351	B.1.617.2	P.1
WHO Label	Alpha [30]	Beta [13]	Delta [62]	Gamma [65]
Country First Detected	England [30]	South Africa [13]	India [56]	Brazil [65]
First Detected	September 2020	October 2020	December 2020	December 2020
Spike mutations	69–70HV and 144Y deletions, N501Y, D614G, A570D, P681H, T716I, S982A, D1118H [33] E484K, S494P, and K1191N (found in some sequences) [35]	L242–244 deletions, A701V, D215G, D80A, D614G, E484K, K417N, N501Y, R246I, L18F [50]	156–157 deletions, D614G, D950N, L452R, T19R, T478K, P681R, R158GG142D (Found in some) [62]	K417T, E484K, N501Y, L18F, T20N, P26S, D138Y, R190S, D614G, H655Y, V1176F, T1027I [67]
Transmissibility	43–82% more transmissible [30]	50% more transmissible [53]	60% more transmissible [61]	Some studies reported 1.7–2.5 times more transmissible [65,74,75]
Viral Load	High [23,36] No difference [38]	High [49]	High in animal model [63]	High in reinfection case [15]
Duration of Infection	Long [26]	N/A	N/A	N/A
Hospitalization	High [32,39]	High [32]	High [61]	High [32,74]
Mortality	Increase [25,40]	Increase [52]	N/A	Increase [65]
Severity	No change [38,42,43]	N/A	N/A	N/A
Risk of reinfection	Not higher [42]	High [51,76]	N/A	6.4% [74]
Resistant to antibody neutralization	Resistant to most ^a^ mAbs directed against ^b^ NTD and slightly resistant to some mAbs directed against the ^c^ RBD [44]	Resistant to most mAbs directed against NTD and many mAbs directed against the RBD [44]	N/A	Resistant to some mAbs directed against RBD [69]
Resistance against convalescent plasma and sera	Less resistant [11,44]	More resistant [11,44,77]	N/A	Less resistant than B.1.351 [44,78]
Vaccine efficacy	Minimal impact [21,22]	Decrease for Pfizer [33], Moderna [79], Novavax, Johnson & Johnson [80,81,82], AstraZeneca [82,83]	2 doses of Pfizer [84,85,86] or AstraZeneca [84] is still protective	Decrease for CoronaVac [87]

^a^ mAbs: monoclonal antibodies. ^b^ NTD: N-Terminal domain. ^c^ RBD: receptor binding domain.

## 3. Pathophysiology of SARS-CoV-2 Variants

The emergence of new variants is raising concerns globally due to their high transmissibility. Their extent of mutations in the spike gene and the recurrent deletions in four discrete regions of the NTD provides resistance to neutralizing antibodies, resulting in selective pressure and antigenic change, ruining the protection provided by mAb therapies and vaccines and leading to convergent evolution. The selective pressure probably led to variants with fitness advantages in infection, transmission efficacy, and replication. As shown in a study, the potential evasion from antibodies may be due to a byproduct of immune pressure in an individual during chronic SARS-Cov-2 [28]. The convergent evolution occurs to evade the common selective pressure, as demonstrated by an onward community transmission where phylogenetic analysis showed independent distinct branches of diverse origins [88]. Although SARS-CoV-2 encodes an exoribonuclease harboring a proofreading function during replication, the virus continues to have mutations in its genome [89]. VOCs have now spread globally and have become the dominant strain in the affected areas. This accelerated evolution of the SARS-CoV-2 genomic variation has led to viral sequence surveillance, including by the CDC in the US and COG-UK in the UK and GISAID [90].

Although the B.1.351 variant surfaces independently of the B.1.1.7, they do share some similarities [91]. Prior to the rise in dominance of the B.1.1.7 and B.1.351 strains possessing both 501Y and deletions, a considerable population of N501Y-only mutants and NTD deletion-only mutants was found in both the UK and South Africa [77]. A common similarity in B.1.1.7, B.1.351, B.1.617.2, and P.1 is that they share significant genetic divergence, with each having > eight missense mutations in the spike protein [13,92,93]. They also have mutations in the NTD. B.1.1.7 has two deletions (Δ69–70 and Δ144); B.1.351 has four amino acid changes and one deletion (Δ242–244) [67,77]; B.1.617.2 has a deletion (Δ156–157) [62]; and P.1 has six amino acid changes [67]. Mutations at the NTD could be concerning as it is targeted by some highly potent neutralizing antibodies [94]. The deletions disrupt the binding sites of neutralizing anti-NTD antibodies where anti-NTD mAb 159 failed to carry out neutralization [77]. Additionally, both B.1.351 and P.1 have the same three residues with mutations: N501Y, E4848K, and K417N/T [65]. B.1.351 and P.1 are also linked with an accelerated surge in cases in locations where previous attack rates are high [55]. Lastly, all four VOC variants have several mutations across the genome, including a few in the spike protein and its RBD [66,95,96], including a deletion in ORF1b (del11288–11296 (3675–3677 SGF)) [55]. These mutations impact SARS-CoV-2 in binding affinity, cell entry, infectivity, neutralization, and vaccine efficacy. The summarized information regarding the key mutations of the VOC and their implications has been tabulated in Table 2.

### 3.1. Entry of SARS-CoV-2: Spike Glycoprotein/ACE2

Every SARS-CoV-2 virus has around 90 homo-trimeric spike receptors in its membrane that differ in height between 9 nm and 12 nm [97]. Viral receptors define tropism, spread, and the virus’s ability to evade the immune system [98]. The entry of SARS-CoV-2 into host cells is mediated by the binding of the viral surface spike (S) protein to the host cell receptor angiotensin-converting enzyme 2 (ACE2) via the receptor-binding domain (RBD) of S protein consisting of a core of a receptor-binding motif (RBM) that interacts with ACE2 [99]. In the lungs and the upper airways, the human ACE2 expression is limited to a particular type of cells, such as type 2 alveolar cells in the lungs and goblet cells in the nasal mucosa [100].

The RBD of the spike glycoprotein is the key antigenic determinant and acts as the primary target of neutralizing antibodies after SARS-CoV-2 infection. It has two subunits: S1 binds to SARS-CoV-2 cellular receptor ACE2, while S2 assists fusion between the cellular and viral membrane [101]. Hence, mutations on the RBD are concerning. Furthermore, various naturally selected mutations in the RBM have been associated with human-to-human transmission, pathogenesis, infectivity, and immune escape [102]. Hence, the current vaccines developed based on earlier strains are deemed to be less effective or ineffective. The ACE2 interaction surface is at the tip of the RBD and consists of a small 25 amino acid patch that allows binding of potent neutralizing antibodies and viral attachment [99,103]. Ceasing RBD–ACE2 interaction could protect against SARS-CoV-2 infection. Thus, the combinations of several of such mAbs are undergoing trials for the prevention and treatment of SARS-CoV-2 [104].

The ACE2 binding surface could also be blocked by some neutralizing antibodies and could jeopardize immune escape, resulting in the decreased potential of vaccine-acquired or natural immunity in suppressing viral replication. Two drivers are possible for the selective pressure on alterations in the ACE2 interaction surface. First, with SARS-CoV-2 crossing the zoonotic barrier, the evolution of ACE2 interaction surface could occur to allow binding to ACE2 with greater affinity, hence increasing the viral transmission. Second, alterations to the ACE2 interaction surface could decrease vaccination or previous infection protection, resulting in immune escape [77].

### 3.2. Mutations Affect Binding Affinity (N501Y, E484K/E484Q, K417N/T)

B.1.1.7, B.1.351, B.1.617/B.1.617.2, and P.1 have RBD mutations that impact interaction with ACE2 receptors and neutralizing antibodies. Compared with the progenitor strain, the binding affinity of B.1.351 to ACE2 has a 19-fold increase due to the three RBD mutations and D614G [77], while B.1.1.7 has a 7-fold increase in binding affinity [33]. B.1.1.7, B.1.351, and P.1 variants have acquired mutations in the ACE2 interaction surface of the RBD sharing the N501Y mutation. In terms of binding affinity, the N501Y mutation has a 10-fold increase in binding affinity to ACE2, which is due to solid aromatic interactions of π stacking between Y41 (Tyr41) and Y501 (Tyr501) and to the formation of two hydrogen bonds with K353 (Lys353) and D38 (Asp38) [105]. This stabilizes K353 on ACE2, a position that allows SARS-CoV-2 to be differentiated from severe acute respiratory syndrome coronavirus (SARS-CoV) and allows for greater binding affinity to ACE2 [99,106]. Additionally, Y501 destabilizes RBD-down conformation, enhancing the D614G effect of more open RBDs [107]. However, the hydrogen bond between residue Y501 and D38 applies only to B.1.1.7 and B.1.351 [108]. As N501Y mutation allows for the increased affinity for ACE2 [54,108], it could also be the cause of increased transmissibility of B.1.1.7 [92,109]. Nevertheless, it has also been proposed that the N501Y mutation does not operate alone in terms of causing increased transmissibility, as the N501Y mutation is seen in some other variants that do not spread faster [110].

A mutation at position E484 (Glu484) is present on B.1.351 (E484K), B.1.617 (E484Q), and P.1 (E484K). E484 stabilizes the RBD down confirmation via interaction with F490 and N343-glycan in neighboring RBD [107]. However, the E484K mutant does not have these interactions, thus favoring RBD-up confirmation due to S1 movements [111]. In naturally occurring SARS-CoV-2 isolates, mutation E484K and E484Q have neutral to mildly advantageous effects on the affinity of RBD for ACE2 [54]. With regard to the progenitor strain and the B.1.1.7 that has an E484K mutation [108], residue E484 interacts with the K31 (Lys31) interaction hotspot residue of ACE2 by disrupting the electrostatic bond (length 3.17–3.19 Å), moderately increasing the binding affinity of RBD to ACE2 [13,54,112]. Concerning B.1.617, a study showed the combination of E484Q and L452R or L452R alone in B.1.617 confers greater binding affinity to ACE2 than variants with only the E484K mutation [113]. For the P.1 variant, a study showed that the E484 residue forms a strong hydrogen bond (length of 2.60 Å) with residue E75 (Glu75) on human ACE2, near enough to form a salt bridge, strengthening the binding affinity [108]. In contrast, a study showed that in B.1.351, the E484K mutation has no significant effect on the binding affinity between SARS-CoV-2 RBD and ACE2 [108,114]. However, this contradicts studies that showed that E484K enhances the binding with ACE2 through a conformational change of the S glycoprotein [115], and another study stating that spike mutations K417N/T and E484K in B.1.351 and P.1 demonstrate a higher affinity for ACE2 [52]. A study revealed that the N501Y/D614G/E484K RBM mutants had an increased binding affinity to ACE2 compared with either K417/D614G/N501Y or N501Y/D614G. This finding by Kim et al. shows that there is a correlation between the increased affinity of E484 for ACE2 and significant conformational change in loop-3 (L3) in the mutant’s RBM, and he proposed that mutation of E484K may impact the stability of ACE2’s binding interface [101]. However, this proposal was rebutted by Hoffman et al., who found B.1.1.7, B.1.351, and P.1 to have no significant difference in spike protein stability and entry kinetics as compared with the progenitor isolate with D614G exchange [116]. Nonetheless, E484K mutations may provide the virus with new features for antibody evasion [117].

Mutation at K417 may impact the affinity in B.1.351 (K417N) and P.1 (K417T) [106,118,119]. Residue K417 is an ACE2-interacting residue that forms a salt bridge with D30 (Asp30) of ACE2 across the central contact region [106,118]. This salt bridge is important for the stability of the RBD–ACE2 complex [120]. However, K417N and K417T in B.1.351 and P.1 have an unfavorable contribution in binding affinity. The distance between these residues and ACE2 is great, resulting in an insignificant molecular interface. This leads B.1.351 and P.1 to lose the salt bridge between position 417 on progenitor RBD and D30, as electrostatic attraction forms when the distance between two amino acids with opposite charges are <4 Å apart [16]. The K417N/T mutations are unfavorable for RBD–ACE2 complex formation [108,121]. A study also described the K417N mutation as having a lower probability of contact [115]. Hence, K417N//T mutations appear to have a moderate impact on the binding affinity of RBD–ACE2 [108]. In addition, although a mutation in K417N can destabilize the RBD-down conformation, increasing the tendency for open configuration [107], it is uncertain whether it applies similarly to the K417T mutation in P.1. A study also found K417N to be associated with N501Y, affecting binding affinity and antibody binding [122]. This could probably counterbalance the unfavorable effect of K417N/T mutations on RBD–ACE2 complex formation, consistent with another study [121]. Thus, the K417N/T mutation may not significantly impact the binding affinity between the RBM and ACE2.

### 3.3. Mutations Increase Cell Entry and Infectivity (Δ69–70, A570D, S982A, D614G, E484K/Q, K417N/T, P681H/R, L452R) 

Regarding the infectivity of B.1.1.7, the deletion of Δ69–70 in the spike protein is concerning, as it arose independently in a few lineages [30,109]. Devies and colleagues revealed that with the N501Y mutation maintained and Δ69–70 reversed, pseudotyped viruses lost considerable infectivity [24]. Furthermore, Δ69–70 deletion could increase infectivity by 2-fold over a single round of infection [109]. Thus, the findings prove that Δ69–70 does affect the surge in infectivity of the B.1.1.7 VOC.

The spike protein directs cell–cell fusion, leading to syncytia formation, probably responsible for viral pathogenesis [123]. In terms of the effect of spike protein mutation on cell fusion, a study showed that compared with the progenitor isolate with D614G exchange, B.1.1.7 has similar efficiency in cell fusion, whereas B.1.351 and P.1 have reduced efficiency [116]. There are three evident mutations of B.1.1.7 on the interface between trimeric protomer subunits that decrease intermolecular binding affinity. In the progenitor strain, between every single chain of SARS-CoV-2 spike glycoprotein—at A570, D614, and S982—there are intermolecular interactions. In B.1.1.7, these amino acids at the three sites mentioned undergo substitution, causing dynamic virus processes to be enhanced, including cleavage of spike protein, structural rearrangement, and mechanism of host cell fusion. The A570D substitution prompts a steric clash with the backbone amide of K964.

In contrast, D614G substitution causes the interface of spike protein subunits in the trimer to form a distinctive cavity, whereas S982A substitution lacks intermolecular hydrogen bonding potential between the spike protein subunits at this site. Thus, this proposes that mutation in B.1.1.7 boosts the affinity of SARS-CoV-2 to ACE2 and that substitutions of A570D, D614G, and S982A possibly enhance the dynamic viral fusion mechanism via a reduction in the intermolecular stability of spike protein subunits [34]. As mentioned earlier, this contradicts Hoffman et al., who found B.1.1.7, B.1.351, and P.1 have no significant difference in spike protein stability and entry kinetics compared with the progenitor isolate with D614G exchange [116].

The D614G mutation is present in all four VOC variants. D614G demonstrates increased human host infectivity and higher transmission efficiency to SARS-CoV-2 [6,95]. D614G has also been shown to strengthen cleavage efficiency by substituting spike conformational diversity [101,124,125]. The B.1.351 infected cells have 2.5 times greater infectivity than D614G, and mutations on the RBM of the RBD may induce changes that increase the binding affinity of the spike protein to ACE2 compared with the progenitor strain [101]. Kim et al. suggested that K417N and E484K substitution contribute to the B.1.351 lineage becoming more efficient at cleaving S1/S2 forms of spike protein to stimulate cell fusion, allowing for increased cell entry efficiency [101].

Mutation at the P681 position also contributes to SARS-CoV-2 transmission and infection [126,127]. It is located adjacent to the RRfAR proprotein convertase motif, a characteristic of high pathogenesis (progenitor strain: PRRAR [99]; B.1.1.7: HRRAR) [34]. Mutation at position P618 is present in B.1.617/B.1.617.2 (P618R) and B.1.1.7 (P618H). This mutation occurs outside the RBD but within the spike and sits adjacent to the S1/S2 furin cleavage site [112]. The S1/S2 furin cleavage site of SARS-CoV-2 is not detected in closely related coronaviruses and has been demonstrated to boost entry into respiratory epithelial cells and transmission in animal models [126,127,128]. Regarding B.1.1.7, at site 681, the cleavage of endosomal S1/S2 furin and other proteases occurs in an acidic environment, forming protonated histidine and affecting cleavage rate. The 681 cleavage occurs under the mediation of S2 heptad repeat domains, resulting in structural rearrangement and host cell fusion, allowing cell entry [34]. However, the study stated that although this mutation causes a slight increase in S1/S2 cleavage, it does not significantly affect viral fitness [129]. For B.1.617, the combination of L452R and P618R contributes to an increased binding and subsequent cleavage of the spike protein, which enhances membrane fusion and systemic infection, likely leading to an enhanced transmission [112]. It is observed that the combination of E484Q and L452R mutation in B.1.617 confers a greater binding affinity to ACE2, possibly contributing to immune evasion [54]. Studies have shown that L452R increases infectivity by stabilizing the S glycoprotein and ACE2 interaction [130,131,132]. Mutation of the L452R causes huge increments in free energy at the RBD and ACE2 binding complex, which results in stronger cell–virus attachment and greater infectivity [131,133]. Another study also shows that the L452R mutation could evade the human leukocyte antigen (HLA)-24 limited cellular immunity, boost viral infectivity, and possibly stimulate viral replication [134]. Although we know roughly how these individual mutations in B.1.617 impact the disease, the combined effect is not yet known. Nonetheless, in conclusion, B.1.1.7, B.1.351, and B.1.617/B.1.617.2 variants seem to have greater infectivity than the progenitor strain and to possibly have enhanced SARS-CoV-2 cell entry.

### 3.4. Impact of Mutations in The RBD on Plasma Binding and Neutralization (K417N/T, N501Y, E484K/Q, L452R)

There is a vital contribution of RBD-binding antibodies to plasma neutralization [72]. RBD-binding antibodies make up a relatively moderate amount of all spike-binding IgG plasma antibodies in naturally infected patients. This is in line with studies showing that <50% of mAbs and spike-reactive B cells bind to RBD [135,136,137,138]. Mutations mainly influence the plasma antibody binding at a few dominant epitopes on the RBD [72]. Mutations that reduce binding are found in one of three discrete regions of the RBD: the surface patch in the core receptor binding motif (RBM), receptor binding ridge within the RBM, and loop in the RBM opposite the ridge [72]. Plasma antibody neutralization is most affected by mutations on the loop in the RBM formed by residues 443–450, followed by mutations on structurally adjacent sites in the receptor-binding ridge of RBD (F490, F486, G485, F456, and L455), and lastly in the core of RBD distal from the RBM [72]. A few other studies have also demonstrated the effect of plasma neutralization to be in the epitope focusing in the 443–450 loop or around E484 [51,71,117,139,140].

Mutations change the antigenic surfaces on the spike protein, resulting in almost total resistance to neutralization by specific mAbs and polyclonal antibodies [141,142,143]. Mutations of the K417N/T, N501Y, and E484K are associated with immune evasion and are found in the B.1.351 and P.1 variants. The amino acid substitution in the RBD (N501Y, E484K, and K417N/T) are accountable for the loss of mAb binding. While these mutations are nominally in different epitopes, their overlapping nature may allow them to be close enough that >1 may directly influence the binding of any one antibody. Additionally, allosteric effects may be present, allowing effects to extend over some distance [77].

The most potent SARS-CoV-2 neutralizing antibodies are the mAbs targeting the RBD. A study showed that B.1.351 is resistant to a crucial group of potent mAbs targeting the RBD. For instance, activities of COV2-2196 and Brii-198 dropped 14.6 times and 6.3 times against the B.1.351 variant, while COV2-2130 and Brii-196 are unaffected by both B.1.1.7 and B.1.351. Moreover, the activity of REGN10933 (casirivimab) is compromised, while LY-CoV555 (bamlanivimab) alone and in combination with CB6 is inactive against B.1.351 [44]. This is consistent with another study demonstrating B.1.351 and P.1 are only partially inhibited by REGN10933 and are utterly resistant to LY-CoV16 (Lilly) [67], LY-CoV555, and REGN10989. However, the combination of REGN10933 and REGN10987 (imdevimab) within an antibody cocktail with emergency use authorization (EUA) (REGN-COV2) reestablishes the inhibition against B.1.1.7, B.1.351, and P.1 [116]. A study also found that the Adagio antibodies ADG10, ADG20, and ADG30 were 100% neutralized by B.1.1.7, B.1.351, and P.1, and in fact, ADG30 has a slight increased neutralization of P.1 [67]. Thus, it can be seen that B.1.351 and P.1 are resistant to only a certain group of antibodies targeting the RBD and that single antibodies with EUA may have no protection (bamlanivimab) or partial protection (casirivimab) against B.1.351 and P.1 variants [116]. It is also observed that REGN10933 and LY-CoV555 evasion are associated with residue 484–486 and are affected by mutation E484K, whereas LY-CoV16 is affected by changes at 417 and 501 [67], which is consistent with other studies showing that LY-CoV555 is sensitive to mutation at residue 484 and that LY-CoV16 is sensitive to changes at 417 [143,144].

K417N/T is one of the significant mutations present in B.1.351 and P.1, which has been shown to escape the neutralization by mAbs [71,143,145]. There are four major classes of neutralizing antibodies to the RBD [146]. Of these, antibodies in classes 1 and 2 seem to be primarily present during SARS-CoV-2 infection, and their epitopes directly overlap the ACE2 binding site [147]. Class 1 antibodies have a VH3-53 restricted mode of recognition centered around K417 spike residue. Mutation K417N stops crucial interactions with class 1 neutralizing antibodies and possibly has a role in immune evasion [117,142,143,148]. A study showed that K417N substitution in B.1.351 mediates the total loss of 910-30 and CB6, while another study showed E484K and K417N mediate the significant drop in the activity of REGN10933 [44,145]. There are published reports demonstrating that the E484K and K417N substitutions are related to the evasion of mAbs [13,72,117,142,143]. A few studies proposed that the combination of N501Y+E484K+K417N found in B.1.351 could result in a more significant reduction in neutralization compared with any of these mutations alone [51,70,71]. Although mutation of K417N has been demonstrated to escape neutralization by some mAbs [143,145], a study conducted by Greaney et al. showed that only a few samples were modestly affected by binding due to mutation at site 417 [72].

The residue 501 is located outside of the major neutralizing epitope clusters in the RBD [141]. It is present in the epitope defined by the 443–450 loop. [72] The mutation from asparagine to tyrosine does not cause any widespread conformational shifts. Hence, there should only be minimal effects on the neutralization of the RBD-binding antibodies against the B.1.1.7 [141]. Even so, the mutation of N501Y may still contribute to the escape of some class 1 neutralizing antibodies [117,142,143,148]. For instance, a study showed that N501Y substitution mediates a drop in the activity of the mAb 910-30 against B.1.1.7 [44]. Another study found that the combination of N501Y and K417N enhanced the binding with ACE2 while significantly decreasing the binding with antibodies [122]. Nonetheless, reports from studies have shown that the antigenic effects of N501Y are limited to a small number of mAbs with no significant impact on the neutralizing activity of convalescent plasma or sera from vaccinated individuals [44,46,79,149]. This statement is somewhat consistent with Greaney et al., who also found that although mutations at N501 have a modest effect on binding by few mAbs [143,145], this mutation did not strongly affect binding by plasma from their studies [72]. This could be because N501Y is positioned at the edge of the ACE2/spike protein interface. Recognition of SARS-CoV-2 peptides by T cells are in the form of multiple HLA molecules, hence possibly does not drastically affect the overall function of polyclonal T cell responsiveness [34].

Mutation of the E484 impacts antibody resistance of B.1.1.7+E484K, B.1.351, B.1.617, and P.1. As compared with the previous D614G spike protein, the E484K mutation present in B.1.351 and P.1 spike proteins has been demonstrated to result in partial resistance to neutralization [44,46,51,78,150,151]. Additionally, E484K mutations generally result in the most significant reductions in binding and neutralization [72]. This could be because antibodies using heavy chain germline genes common among anti-SARS-CoV-2 RBD antibodies IGHV3-66 and IGHV3-53 often target E484 [117,143,146,152,153,154]. In contrast with class 1 antibodies that bind to K417, class 2 antibodies bind to E484 spike residue. Studies show that the E484K mutation causes resistance to neutralizing antibodies in class 2 and convalescent sera; hence, E484K may be a dominant neutralizing epitope [117,142,143,148]. This E484Q mutation in B.1.617 also plays a role in immune evasion. It is associated with reduced convalescent serum neutralization, neutralization of antibodies, and the ability to reinfect individuals who have not been infected by these mutated variants [142,143]. Although some plasmas are unaffected by mutation at E484, many experiments showed that it reduces the neutralization potency of some human plasma by over 10-fold [117,140,142]. According to Greaney et al., the neutralization titer of several plasma are reduced against viruses pseudotyped with E484 mutations. Nonetheless, a single mutation cannot revoke neutralization for the plasma samples, as mutations at other plasma antibody epitopes can also contribute to antigenic impacts [72].

On the other hand, the intramolecular interaction in the progenitor strain suggests that L452 residue plays a role in the hydrophobic interaction with L492 connected to F490 via another hydrophobic bond. A hydrophobic patch is formed on the RBD surface as a result of these residue interactions. However, mutations on the L452R ceases this hydrophobic interaction with the L492 of the RBD [112]. According to Cherian et al., the combined effect of L452R and E484Q found in the B.1.617 variant disrupts the interfacial interactions of spike RBD with specific neutralizing antibodies. In their study, mAb REGN10933 interacts with RBD via two hydrogen bonds between E484 of RBD and antibodies S56 and Y53. Y453 of RBD is connected with D31 of the antibody via a hydrogen bond. However, the E484Q mutation disrupts the two hydrogen bonds with Y53 and S56. In addition, both L452 and E484 have a direct connection with mAb P2B-2F6. Hence, mutations on both L452 and E484 positions could reduce the ability of mAb REGN10933 and P2B-2F6 to bind variant strains [112].

### 3.5. Impact of RBD Mutations on Neutralizing Activity of Convalescent Plasma or Sera (L452R, E484K/Q)

Several studies showed that RBD-binding antibodies account for most of the plasma neutralizing activity of most convalescent human plasma and sera [147,155]. Production of neutralizing antibodies induced by SARS-CoV-2 infection are thought to play a part in protection from reinfection [156,157]. Hence, it is crucial to know if VOCs are effectively neutralized by antibody responses in convalescent SARS-CoV-2 patients [116]. 

The mutation in B.1.617, L452R, appears to reduce the sensitivity to certain antibodies and human convalescent sera [139,158]. Studies on neutralization revealed that antibodies from previously infected SARS-CoV-2 patients bind to the L452R-carrying pseudoviruses to a lesser extent. The convalescent plasma samples escaped neutralization [132,142]. The effect on neutralizing antibody binding is speculated by Deng et al. to result from L452 mutation in a hydrophobic pocket, inducing conformational changes in the RBD. They also suspect that the emergence of L452R could be driven by the immune selection pressure from earlier exposed populations, as there is more than a 4-fold reduction in neutralizing antibody titers in convalescent plasma [132].

The mutation at site E484 on B.1.1.7+E484K, B.1.351, and P.1 demonstrated decreased neutralization by both mAbs and human plasma or sera [69,71,117,140,143,145]. In a study conducted by Wang et al., the mutation E484K results in relative resistance, as the convalescent plasma from SARS-CoV-2 patients collected earlier in the pandemic showed that against B.1.1.7 there is no significant effect in the neutralizing activity. Still, against B.1.351, there is a great reduction [13,44]. Out of 20 patients, only 4 plasma samples maintained neutralizing activity similar to that against the wild type. It was also found that three samples consisted of neutralizing antibodies that were unaffected by the spike mutations on both B.1.1.7 and B.1.351 variants [44]. Studies have found that the decrease in neutralization activity by convalescent plasma and human sera in P.1 is not as huge as it is in B.1.351 [44,78]. (Table 1) Mutations mainly influence plasma antibody binding at a few dominant epitopes on the RBD [72]. Hence, the relative resistance shown indicates that the E484K mutation in the RBM is located in an immunodominant epitope known by sera of the studied vaccinated individuals [44].

Consequently, concerns about reinfections are raised, which have also been proposed by other studies [51,70]. The finding that the neutralizing activity of sera from vaccinated individuals against B.1.1.7 is mostly complete without adverse effects from current vaccines is also consistent with several other studies [79,159,160]. For example, B.1.1.7 was still neutralized by sera and antibodies induced by BNT162b2-vaccinated individuals [46,160]. Research using serum samples from participants in Moderna’s vaccine (mRNA-1273) phase 1 trial also showed B.1.1.7 had no significant effect on neutralization [79]. On the contrary, a study evaluated the neutralization of sera collected from COVID-19 patients and vaccinees with two doses of Bharat Biotech’s Covaxin (BBV152) against B.1.617.2 and B.1.351. The findings demonstrated a reduction in neutralization titer and that the Covaxin (BBV152) vaccine had a protective response against B.1351 and B.1.617.2 variants [161].

B.1.351 has an average 13-fold reduction in the neutralization by serum from convalescent plasma [77]. There is also a reduction in the neutralization by serum of vaccinated individuals. Those immunized with Oxford–AstraZeneca (AZD1222) had a 9-fold reduction, whereas those vaccinated with Pfizer–BioNTech (BNT162b2) had a 7.6-fold reduction [77]. Another study reported that for all three variants of B.1.351, the neutralization after vaccination with BNT162b2 showed >70-fold reduction, with B.1.351-v2 showing the most significant reduction of ≥90-fold, all as a result of the three RBD mutations [78]. Studies also found a 10.3- to 12.4-fold loss in activity against B.1.351, greater than those using mutant pseudoviruses [71,79,150]. This is worrying, as reports have also shown a significant decrease in the efficacy of the Johnson & Johnson and Novavax vaccines in South Africa [80,81].

On the other hand, the loss of neutralizing activity of convalescent plasma and vaccine sera against P.1 is less than that of B.1.351 [44,78]. In a study using the Victoria strain as a comparison, neutralization of P.1 by convalescent plasma showed a 3.1-fold reduction in neutralizing capacity of immune serum similar to B.1.1.7, but less severe than B.1.351, which had 13-fold reduction [33,67,77]. Another study showed neutralization against P.1 involving serum samples from 10 individuals who received BNT162b2 [17] and 12 who received mRNA-1273 [162]; the study showed that the magnitude of the loss was 3.8- to 4.8-fold [69], which was not as severe as the magnitude of the loss of 10.3- to 12.4-fold seen against B.1.351 [44]. Research using serum samples from participants in the mRNA-1273 phase 1 trial showed reductions by a factor of between 2.3–6.4 in titers of neutralizing antibodies for variants B.1.1.7+E484K, B.1.351, and P.1 [79].

### 3.6. Impact of Deletions in the NTD (Δ69–70, ΔY144 Deletion, ΔL242–Δ244, and/or R246I) 

Recurrent deletions in four discrete regions of the NTD caused resistance to neutralizing antibodies, resulting in selective pressure and antigenic change [28]. Patients treated with convalescent plasma had deletions in the NTD, thus proposing a fitness improvement via the host’s immune response evasion [88]. Hence, in addition to RBD, antibodies may act on other spikes, as shown by the effects of neutralization of plasma antibody due to mutations and deletions in the NTD [28,29,135,140,163]. With regard to the NTD, B.1.1.7 has Δ69-70 and Δ144 deletion, whereas B.1.351 has Δ242-Δ244 deletion. Wang et al. demonstrated that both B.1.1.7 and B.1.351 are resistant to neutralization by mAb directed against the NTD supersite [44]. A study showed Δ69–70 deletion modifies loop 2 (69–76aa), pulling it nearer to the NTD [29]. The ∆Y144 and the ∆L242/L244 deletions show a loss of binding ability with neutralizing antibodies [28,44]. Two studies found that ΔY144 deletion in loop N3 of the supersite confers the resistance of B.1.1.7 to a majority of NTD-directed mAbs, whereas one study found Δ242–Δ244 and/or R246I conferring the resistance of B.1.351 [34,44]. These amino acid residues all happen to fall within the NTD supersite [94,103].

There is a trend of recurrent deletions found in four discrete regions of the NTD, providing resistance to antibody neutralization. The study by McCarthy demonstrated that 1108 out of 146,795 sequences obtained from GISAID had S gene deletions, with 90% occupying the four discrete sites (recurrent deletion regions 1–4 i.e., RDRs) within the NTD [28]. The RDRs occupied different regions of the NTD with other deletions. It was shown that when the spike protein carries the deletions Δ69–70 + Δ144/145 (RDR1 + 2), Δ141–145 or Δ144/145 or Δ146 in RDR2, and Δ243–244 in RDR4, 4A8 neutralizing mAbs did not bind to the spike protein, but the binding occurred for deletions Δ69/70 alone in RDR1 and Δ210 in RDR3 [28]. Although 4A8 mAb did not neutralize the virus, the virus was neutralized by polyclonal antiserum from other convalescent patients, consistent with another study [28,46]. This suggests that deletions in the NTD alone do not stop neutralization by a group of antibodies targeting different parts of the S epitope. Furthermore, it is demonstrated that identical or similar recurrent deletions that modify positions 144/145 and 243–244 in the spike glycoprotein disrupt binding of antibody 4A8, defining an immunodominant epitope within the NTD [28].

With regard to P.1, although it does not have NTD deletions, it does have point mutations that may confer similar functional properties [77]. Furthermore, B.1.351 and P.1 have a matching key mutation at the RBD but have inconsistencies in their neutralization susceptibility to polyclonal sera or plasma. This suggests that NTD mutations could impact SARS-CoV-2 susceptibility to antibody neutralization [69].

**Table 2:** Key mutations of VOCs and their implications.

Key Mutations	Implications	References
D614G	Increases human host infectivity and transmission efficiency.	[6,95]
	Strengthens cleavage efficiency by substituting spike conformational diversity.	[101,124,125]
Δ69–70 deletion	Modifies loop 2 (69–76aa), pulling it nearer to the ^a^ NTD.	[29]
	Increases infectivity by 2-fold over a single round of infection.	[109]
ΔY144 deletion	Loss of binding ability with neutralizing antibodies.	[28,44]
ΔL242–Δ244	Loss of binding ability with neutralizing antibodies.	[28,44]
A570D, D614G and S982A	Possibly enhances dynamic viral fusion mechanism via the reduction in intermolecular stability of spike protein subunits.	[34]
	However, contradicts Hoffman et al., who found that B.1.1.7, B.1.351, and P.1 had no significant difference in spike protein stability and entry kinetics compared with the progenitor isolate with D614G exchange.	[116]
N501Y	Increases binding affinity to ^b^ ACE2 due to solid aromatic interactions of π stacking between Y41 (Tyr41) and Y501 (Tyr501), and forming two hydrogen bonds with K353 (Lys353) and D38 (Asp38).	[54,99,105,106,108]
	Could be the cause for increased transmissibility of B.1.1.7 and B.1.351.	[52,92,109]
	Contributes to the escape of some class 1 neutralizing antibodies.	[117,142,143,148]
	Antigenic effects limited to a small number of ^c^ mAbs, with no significant impact on the neutralizing activity of convalescent plasma or sera from vaccinated individuals.	[44,46,79,149]
	Does not drastically affect the overall function of polyclonal T cell responsiveness.	[34]
E484K/Q	Mutation E484K and E484Q have neutral to mildly advantageous effects on the affinity of ^d^ RBD for ACE2.	[54]
	**E484K:** Favor RBD-up confirmation due to S1 movements opposite of normal E484, which stabilizes the RBD-down confirmation.	[107,111]
	In progenitor and B.1.1.7+E484K strains, it disrupts the electrostatic bond, increasing the binding affinity of RBD to ACE2 moderately.	[13,54,112]
	In P.1, it forms a strong hydrogen bond with residue E75 (Glu75) on human ACE2, near enough to form a salt bridge, strengthening the binding affinity.	[108]
	Results in partial resistance to neutralization.	[44,46,51,78,150,151]
	Causes resistance to neutralizing antibodies in class 2 and convalescent sera.	[117,142,143,148]
	A few studies found no significant effect on the binding affinity between SARS-CoV- 2 RBD and ACE2.	[108,114]
	**E484Q:** Associated with lower convalescent serum neutralization, neutralization of antibodies, and the ability to reinfect individuals who had not been infected by these mutated variants.	[142,143]
K417N/T	Unfavorable for RBD–ACE2 complex formation.	[108,121]
	Moderate impact on the binding affinity of RBD–ACE2.	[108]
	Escapes neutralization by mAbs.	[71,143,145]
	**K417N:** Destabilizes the RBD-down conformation; increases the tendency for open configuration.	[107]
	Stops crucial interactions with class 1 neutralizing antibodies and possibly has a role in immune evasion.	[117,142,143,148]
P681H/R	Causes structural rearrangement and host cell fusion, allowing cell entry.	[34]
	Contributes to SARS-CoV-2 transmission and infection.	[126,127]
	Causes slight ↑ in S1/S2 cleavage, but does not significantly affect viral fitness.	[129]
L452R	Increases infectivity by stabilizing the ^e^ S glycoprotein and ACE2 interaction.	[130,131,132]
	Causes huge increase in free energy at the RBD and ACE2 binding complex, resulting in stronger cell–virus attachment and increased infectivity.	[131,133]
	Could evade the human leukocyte antigen (HLA)-24 limited cellular immunity, boost viral infectivity, and possibly stimulate viral replication.	[134]
	Can decrease the sensitivity to a few antibodies and human convalescent sera.	[139,158]
N501Y + E484K + K417N	More significant decrease in neutralization compared with any of these mutations alone.	[51,70,71]

^a^ NTD: N-terminal domain. ^b^ ACE2: angiotensin-converting enzyme 2. ^c^ mAb: monoclonal antibody. ^d^ RBD: receptor-binding domain. ^e^ S glycoprotein: spike glycoprotein.

## 4. Variants of Concern (VOCs) Impact on Vaccine Efficacy

Vaccines activate the immune response upon binding to the spike protein. They aim to induce neutralizing antibodies and possibly induce cytotoxic T lymphocytes [164]. The neutralizing antibodies target the S1 RBD, S1 NTD, or S2. These antibodies block RBD from binding the ACE2 receptor and prevent fusion of the S2 membrane, preventing viral infection [165]. Vaccination can be targeted at several sites of the SARS-CoV-2 surface, including unexposed nucleocapsid N, matrix protein M, envelope protein E, and the envelope spike protein [166]. The antigen of choice for a vaccine is the spike protein: It is linked with high neutralizing antibody response, it has been proven pre-clinically against SARS-CoV and Middle East respiratory syndrome coronavirus (MERS-CoV), and it has structural and sequence conservation similarities with SARS-CoV-2 [164,166]. Mutations on the RBD are responsible for most of the escape from vaccine-induced neutralization [78]. Hence, the emergence of new variants due to mutations on the spike protein could affect vaccine efficacy. Currently, a few vaccines have been developed and available for emergency use, including Pfizer–BioNTech, Oxford–AstraZeneca, Moderna, Sinovac, Novavax, Johnson & Johnson’s Janssen, CanSino Biologics, and Sputnik V.

On 31 December 2020, the World Health Organization (WHO) listed the Comirnaty COVID-19 mRNA vaccine for emergency use, making the Pfizer–BioNTech vaccine the first to receive emergency validation since the outbreak [167]. However, with emerging new variants, there are concerns that the mutations in the spike protein may cause conformational changes that would affect vaccine efficacy [46]. Pfizer–BioNTech, Oxford–AstraZeneca, and Moderna SARS-CoV-2 vaccines are based on the defining spike protein within SARS-CoV-2 of the predominant D614G strain detected in March 2020. Although new VOCs detected have mutations within the spike protein that could impact these vaccine efficacies, some researchers believe that as the spike proteins are relatively large, the virus will require many mutations before it can completely evade the vaccine [168]. Hence, the vaccines should still be effective against SARS-CoV-2 [168,169].

On the contrary, there are hypotheses that vaccine efficacy may decrease, as the preliminary trials of Pfizer–BioNTech and Moderna (mRNA) vaccines approved by the FDA were conducted before VOCs were detected and reported in the US [168]. Furthermore, information on the efficacy of the mRNA vaccines against VOCs were from lab studies using serum samples from immunized patients exposed to genetically engineered versions of the variants, measuring them against the neutralizing antibody titers. The studies agree with the hypotheses, showing reduced neutralizing antibody production against the VOC than against the D614G strain [44,143,169]. On the contrary, D614G substitution should not affect vaccine efficacy, as it does not affect vaccine-induced neutralizing antibodies [170]. However, this may not be applicable for some of the new VOCs that have other mutations. With the emergence of new variants and their impact on vaccine efficacy, Pfizer, Novavax, and Moderna are developing booster doses to increase protection against the VOCs [171,172,173].

Regarding B.1.1.7, the susceptibility of neutralizing antibodies has a minimal impact on vaccine efficacy [21,22], and reinfection is not found to be greater than previous strains [42]. Furthermore, based on a study involving the Pfizer–BioNTech vaccine, mutations on N501Y demonstrated no reduction in antibody neutralization efficacy [46]. There is also clinical evidence that Novavax (NVX-CoV2373) and Oxford–AstraZeneca (ChAdOx1 nCoV-19/AZD1222) vaccines protect against B.1.1.7 [47,174], with a study finding the protection from AZD1222 to be 66% [84] (Table 1).

B.1.351, on the other hand, has a greater risk of reinfection, as it causes neutralizing antibodies produced by D614G and different dominant strains to be resistant [51,76]. The E484K substitution in B.1.351 is associated with a high rate of immune escape [44,142,143,175], so theoretically, after an infection or vaccination, the immunity drops [115,175]. Additional vaccine trials conducted in South Africa of the Novavax, AstraZeneca, and Johnson & Johnson’s Janssen vaccines demonstrated that in places where B.1.351 is the dominant strain, the vaccine efficacy is lower [82]. Other studies show that there is a lower rate of vaccine efficacy for Moderna [79] and Pfizer [33] against B.1.351. A study showed a 28% decrease in protection against the development of symptomatic B.1.351 after BNT162b2 vaccination [176]. Although mRNA vaccines (Moderna and Pfizer) and Novavax have decreased immunogenicity, the neutralizing antibody titers produced are within the expected protective range [21]. In addition, although the Johnson & Johnson Janssen vaccine showed lower overall efficacy in South Africa, there was considerably more protection against severe or fatal disease than for mild-to-moderate disease [149,174]. However, AstraZeneca’s vaccine efficacy against B.1.351 was found to be as low as only 0–10% [83] (Table 1).

On the contrary, there is not much evidence available on the B.1.617 vaccine resistance level. Still, an Indian Centre for Medical Research study involving 1300 SARS-CoV-2 positive patients showed that the B.1.617 VOC has a 4.5% reinfection rate [177]. Studies demonstrate that two doses of the BNT162b2 vaccine are protective against B.1.617.2 infection [85,86], with a study stating an efficacy of 88% two weeks after the second dose [84]. A study stated that two doses of AZD1222 provide 60% protection against symptomatic disease from B.1.617.2 [84] (Table 1). Additionally, based on preliminary results, BBV152 manufactured in India has effectively neutralized B.1.617 in both previously infected and previously vaccinated individuals, as mentioned in the previous section [125]. Studies have shown that although there is a reduction in neutralization titers against the B.1.617.2 variant, the neutralization potential is still well established. This is due to the broad coverage epitope of BBV152 that reduces the magnitude of neutralization against emerging variants [161]. However, more studies should be conducted on the efficacy and safety of the BBV152 vaccine.

Lastly, there are not much similar data on vaccine efficacy against P.1. Nonetheless, compared with B.1.351, P.1 is less resistant to vaccine-induced or naturally acquired antibody responses [67]. Since the loss of neutralizing activity of convalescent plasma and vaccine sera against P.1 is less than B.1.351 [44,78], it is hypothesized that the increase in reinfections and the drop in a vaccine efficacy against P.1 is also less significant, consistent with another study [69]. A study suggested that previously infected individuals may only be partially protected against infection involving variants B.1.351 and P.1. The BNT162b2 vaccine may provide less protection against these variants than against the progenitor strain [116]. Additionally, another study found CoronaVac, made by China’s Sinovac Biotech, had a drop in vaccine efficacy from 78% to 50% when against P.1 [87] (Table 1). Preliminary results found that five months after booster immunization with Coronavac, plasma from vaccinated individuals neutralizes P.1 lineage isolates inefficiently [49,178].

## 5. Conclusions

**Figure 1:**
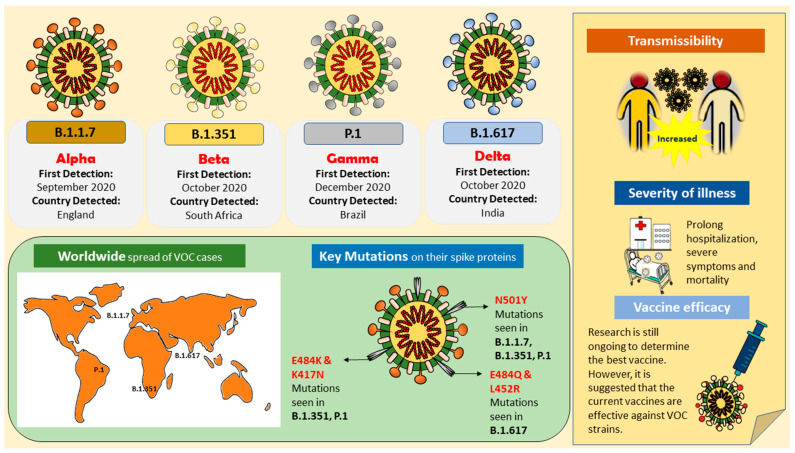
Illustration of variants of concern (VOCs); B.1.1.7 (Alpha), B.1.351 (Beta), B.1.617/B.1.617.2 (Delta), and P.1 (Gamma), their key mutations, and their impact on the public health.

The emergence of VOCs marks the start of SARS-CoV-2 antigenic drift. The B.1.1.7, B.1.351, B.1.617/B.1.617.2, and P.1 variants are more transmissible, and there are data indicating that B.1.1.7, B.1.351, and B.1.617.2 are more infective than the progenitor strain (Figure 1). There is a link between the emergence of these variants and SARS-CoV-2 transmission and epidemic severity. The high transmission rate is a prognostic factor for genomic variations. Thus, outbreaks happen following large gatherings [179]. As viral evolution is present in asymptomatic patients, with the current insufficient isolation period, asymptomatic cases may have higher viral transmission due to impaired viral clearance, which allows viral shedding to occur even >70 days after diagnosis [27]. 

Changes in the spike protein of these variants, especially in the RBD, disrupts the host–virus interaction, affecting binding affinity (N501Y, E484K/E484Q, K417N/T), infectivity and host cell entry (Δ69–70, A570D, D614G, S982A, E484K/Q, K417N/T, P681H/R, L452R), resistance towards neutralizing antibodies (Δ69–70, ΔY144, Δ242–Δ244, and/or R246I) [34,44,67,116], and vaccine efficacy. As currently available vaccines were developed based on the previous D614G strain, the VOCs could have other conformational changes in the spike protein, resulting in findings from various studies showing reduced production of neutralizing antibodies against them [44,143,169]. Furthermore, it is not certain whether antibody responses in convalescent patients could protect against reinfection with the emerging VOCs. Among the variants discussed, as compared with the progenitor strain, B.1.351, P.1 [116], and B.1.617 are resistant to some antibodies used for SARS-CoV-2 treatment [112]. Additionally, B.1.351 and P.1 variants have reduced inhibition by convalescent sera or plasma from patients immunized with vaccine BNT162b2 and AZD1222 [46,67,77,78]. On the contrary, B.1.1.7 is neutralized by sera from BNT162b2 without reducing antibody neutralization efficacy [46,160]. Although the T cell responses may help control SARS-CoV-2 infection, especially in reinfected convalescent individuals, with reduced antibody-mediated neutralization, vaccinated and convalescent individuals may not be completely protected against B.1.351, B.1.617, and P.1 infection. 

Currently, we are unsure how variants of the virus will affect the course of the pandemic, whether it is due to higher transmissibility of the variants, or evasion of the immune system due to waning immunity, or both. Most concerning would be the emergence of viruses with the ability to evade vaccine-induced immunity due to immune pressure [91]. If the virus goes in that direction, it may evade the prophylactic interventions and current vaccines aimed against its spike protein. In conclusion, in spite of the concerns mentioned, vaccination is still crucial for containing this global SARS-CoV-2 pandemic, as vaccines induce neutralizing antibodies and block the viral RBD from binding to the ACE2 receptor. Although there is a reduction in vaccine efficacy against the emerging VOCs, vaccines can still provide a considerable amount of protection, moderating the severity. Additionally, in agreement with another study, mutational changes may not affect T cell responses to spike protein, hence limiting the spread to the lower respiratory tract and preventing severe disease [77]. There also needs to be continuous surveillance of the evolutionary changes of SARS-CoV-2 and how mutations of the spike protein impact immune escape. Lastly, we should continue to practice safety measures to slow the transmission of SARS-CoV-2 and protect ourselves and others from infection [180].
